# Prevalence and associated factors of metabolic-associated fatty liver disease in overweight Finnish children and adolescents

**DOI:** 10.3389/fendo.2023.1090344

**Published:** 2023-06-20

**Authors:** Hanna Riekki, Linnea Aitokari, Laura Kivelä, Siiri Lahti, Pauliina Hiltunen, Nina Vuorela, Heini Huhtala, Timo A. Lakka, Kalle Kurppa

**Affiliations:** ^1^ Tampere Center for Child, Adolescent and Maternal Health Research, Tampere University, Tampere, Finland; ^2^ Celiac Disease Research Center, Tampere University, Tampere, Finland; ^3^ Children’s Hospital and Pediatric Research Center, University of Helsinki and Helsinki University Hospital, Helsinki, Finland; ^4^ Department of Pediatrics, Tampere University Hospital, Tampere, Finland; ^5^ Faculty of Social Sciences, Tampere University, Tampere, Finland; ^6^ Institute of Biomedicine, School of Medicine, University of Eastern Finland, Kuopio, Finland; ^7^ Department of Clinical Physiology and Nuclear Medicine, Kuopio University Hospital, Kuopio, Finland; ^8^ Kuopio Research Institute of Exercise Medicine, Kuopio, Finland; ^9^ University Consortium of Seinäjoki, Seinäjoki, Finland

**Keywords:** alanine aminotransferase, children, diabetes, dyslipidemia, glucose intolerance, MAFLD, NAFLD, obesity

## Abstract

**Introduction:**

Data on the prevalence of pediatric fatty liver disease remain limited, partly due to challenges in diagnosis. A novel concept of metabolic-associated fatty liver disease (MAFLD) makes it possible to establish the diagnosis in overweight children with sufficiently elevated alanine aminotransferase (ALT). We investigated the prevalence, risk factors, and metabolic co-morbidities of MAFLD in a large group of overweight children.

**Methods:**

Data on 703 patients aged 2-16 years examined due to overweight in different levels of healthcare in 2002-2020 were collected retrospectively from patient records. MAFLD was here defined as ALT >2x reference (>44 U/l in girls and >50 U/l in boys) in overweight children according to recently updated definition. Patients with MAFLD and without it were compared, and subgroup analyses were conducted among boys and girls.

**Results:**

Median age was 11.5 years, and 43% were girls. Altogether 11% were overweight, 42% obese and 47% severely obese. Abnormal glucose metabolism was present in 44%, dyslipidemia in 51%, hypertension in 48% and type 2 diabetes (T2D) in 2%. MAFLD prevalence varied between 14-20% in examined years without significant change (p=0.878). The pooled prevalence over the years was 15% (boys 18%, girls 11%; p=0.018), peaking in girls at early puberty and increasing in boys with age and puberty. Associated factors in boys were T2D (OR 7.55, 95% CI 1.23-46.2), postpubertal stage (5.39, 2.26-12.8), increased fasting insulin (3.20, 1.44-7.10), hypertriglyceridemia (2.97, 1.67-5.30), hyperglycemia (2.88, 1.64-5.07), decreased high-density lipoprotein (HDL) cholesterol (2.16, 1.18-3.99), older age (1.28, 1.15-1.42) and higher body-mass-index (1.01, 1.05-1.15), and in girls T2D (18.1, 3.16-103), hypertriglyceridemia (4.28, 1.99-9.21), and decreased HDL (4.06, 1.87-8.79).

**Conclusion:**

Prevalence of MAFLD was 15%, with no statistically significant increase in the 2000s. The condition was associated in general with male gender, puberty stage and disturbances in glucose and lipid metabolism, and higher age and BMI in boys.

## 1 Introduction

Obesity-associated nonalcoholic fatty liver disease (NAFLD) is currently considered to be the most common chronic liver disease in children and adolescents (1–3). However, data on the actual prevalence remain scarce, primarily due to a lack of accurate or practical imaging tools and restricted applicability of liver biopsy in this age group (4). Determining the precise prevalence figures would enable better targeting of lifestyle interventions and limited healthcare resources (5). This issue is particularly important in pediatric patients, who often present with a rapidly progressing condition and may derive great benefit from early treatment (6, 7). Moreover, untreated NAFLD may be an independent risk factor for metabolic and cardiovascular comorbidities (7–10), although actual evidence is again scant.

The soaring prevalence of overweight in children and recognition of the importance of metabolic dysfunction as a contributory factor calls for more biologically meaningful and simplified diagnostics for NAFLD. The recently introduced concept of metabolic-associated fatty liver disease (MAFLD) defines the condition as increased liver adiposity together with the presence of metabolic abnormalities and/or obesity. It is a promising non-invasive diagnostic approach in children, as the presence liver steatosis can be identified based solely on laboratory evidence (11–13). Moreover, the co-existence of another hepatic condition is possible without exhaustive differential diagnostics necessary for the NALFD diagnosis (11, 14). MAFLD might thus offer a more unbiased means to study the prevalence and patient-related risk factors for fatty liver disease, and also to estimate a possible increase of the condition concurrently with overweight and obesity (11, 15).

We aimed to investigate the prevalence, risk factors, and metabolic co-morbidities of MAFLD in a large and well-defined group of overweight children and adolescents examined in different levels of healthcare.

## 2 Materials and methods

### 2.1 Patients and study design

The retrospective cross-sectional study was carried out at Tampere University and Tampere University Hospital. It comprised 1,000 consecutive patients aged 2-16 years who had received an obesity-related ICD 10 diagnosis code E65, E66.0-9 or R63.5 either at the primary care unit of the City of Tampere in 2006-2020 or at Tampere University Hospital in 2002-2020. Their comprehensive medical data was collected retrospectively from systematically maintained patient records. The clinical findings and laboratory data was collected at the time of the first obesity-related investigations in healthcare. Children and adolescents with insufficient data or lacking an alanine aminotransferase (ALT) value and those not deemed overweight after re-evaluation of the anthropometric measurements were excluded. Additionally, altogether 0.17% of the study children had a possibly hepatotoxic medication or a liver-affecting co-morbidity and were consequently excluded for simplicity, leaving 703 participants for the final analyses (**Supplementary Figure 1**).

The study design and collection of patient register data were approved by the City of Tampere Healthcare Services and by Tampere University Hospital according to the national ethical and data processing recommendations. None of the participants were contacted by the research group during the study. The Declaration of Helsinki was strictly followed in all stages.

### 2.2 Data collection and definitions

Age, sex, body mass index (BMI, body weight kg/body height m^2^), waist circumference and puberty stage, blood pressure, presence of acanthosis nigricans and relevant chronic diseases and medications, and use of possibly liver-affecting supplements, herbal products, alcohol, or illicit drugs were recorded at the time of the first obesity-related visit to healthcare.

Severity of overweight was categorized using BMI (kg/m2) and BMI Z-scores or weight-to-height percentages (WH%) as recommended by the International Obesity Task Force expert panel (16). Cutoff values for BMI Z-scores were >1.16 for overweight, >2.11 for obesity, and >2.76 for severe obesity in girls and >0.78, >1.70, and >2.36 in boys. These correspond to BMI values of >25 kg/m2, >30 kg/m2 and >35 kg/m2 at the age of ≥18 years (16, 17). The equivalent values for WH% were 10-20% for overweight, 20-40% for obesity, and >40% for severe obesity in those <7 years, and respectively 20-40%, 40-60%, and >60% in older participants (16, 17). Abnormal waist circumference was defined as >90th percentile for age and sex (18) and hypertension as systolic or diastolic blood pressure >95th percentile (19). Pubertal status was classified by a trained clinician based on Tanner staging as pre-pubertal (stage 1), pubertal (stages 2-4) and postpubertal (stage 5) (20, 21).

The following fasting plasma laboratory values were collected as available: ALT, glucose (reference value <5.6 mmol/l), insulin (prepubertal ≤15 mU/l, pubertal ≤30 mU/l and postpubertal ≤20 mU/l), HOMA-IR (<2.67 for prepubertal and <5.22 for pubertal boys and <2.22 for prepubertal and <3.82 for pubertal girls), total cholesterol (<5.18 mmol/l), high-density lipoprotein (HDL) cholesterol (≥1.04 mmol/l), low-density lipoprotein (LDL) cholesterol (<3.36 mmol/l) and triglycerides (<1.13 before and <1.47 mmol/l after the age of 10 years) (22, 23).

Impaired glucose metabolism was based on a two-hour plasma glucose of 7.8-11.0 mmol/l from a two-hour oral glucose tolerance test (OGTT) or impaired fasting glucose of 5.6-6.9 mmol/L, and type 2 diabetes on a fasting plasma glucose of >6.9 mmol/l or a two-hour plasma glucose of >11.0 mmol/l, as recommended by the American Diabetes Association (24). If OGTT was not available only a fasting glucose value was used.

ALT upper limit of normal (ULN) values of 22 U/l for girls and 25 U/l for boys was applied according to the National Health and Nutrition Examination Survey (NHANES) results and the North American Society of Pediatric Gastroenterology, Hepatology and Nutrition (NASPGHAN) guidelines (4, 25). In addition, an ALT cut-off of 80 U/l was tested separately due to a previously reported increased risk of advanced liver disease (4, 26). MALFD was defined as ALT twice ULN (>44 U/l for girls and >50 U/l for boys) in overweight or obese children (11).

### 2.3 Statistical analyses

Categorical variables are reported as numbers and percentages. Normality of continuous variables were tested with Q-Q Plot and with Kolmogorov-Smirnov and Shapiro-Wilk tests, and were found to be markedly skewed. Therefore, they were reported as medians with lower (Q_1_) and upper (Q_3_) quartiles or with ranges and tested with nonparametric tests. Children with and without MAFLD were compared by Mann-Whitney or Kruskal-Wallis test for continuous variables and by Chi-square or Fisher’s exact test for categorical variables in unadjusted comparisons. Due to the observed differences in age and sex between the two groups, the results were also adjusted by these variables by using binary logistic regression. The risk of having MAFLD according to the characteristics of participants were analyzed separately for boys and girls by using unadjusted logistic regression models and models adjusted for age. The results are reported as odds ratios (OR) with 95% confidence intervals (CI). The prevalence of MAFLD, increased ALT and obesity in children having their first healthcare visit in different timepoints was compared with Chi-square test. Statistical significance was defined as P value <0.05. All analyses were performed using SPSS version 25.0 (Armonk, NY: IBM Corp).

## 3 Results

The median age of the 703 children was 11.5 (range 2.2-16.7) years, 43.0% were girls and 91.0% had Finnish ethnicity. Altogether 10.8% were overweight, 89.2% obese and 46.8% severely obese, and 99.1% had central obesity (**Supplementary Table**). Moreover, 48.4% had hypertension, 29.2% fasting hypertriglyceridemia, 18.2% increased total cholesterol, 20.3% increased LDL cholesterol, 26.0% decreased HDL cholesterol, 81.4% increased HOMA-IR, 41.1% increased fasting insulin, and 35.6% impaired glucose metabolism. Altogether 27.6% had no metabolic abnormalities. Eleven (1.7%) participants fulfilled the criteria for T2D. None of the children or adolescents reported excessive use of supplements or herbal products. Two participants reported occasional alcohol use and in neither of these was the cumulative dose considered hepatotoxic. None were diagnosed with viral hepatitis.

**Figure 1:**
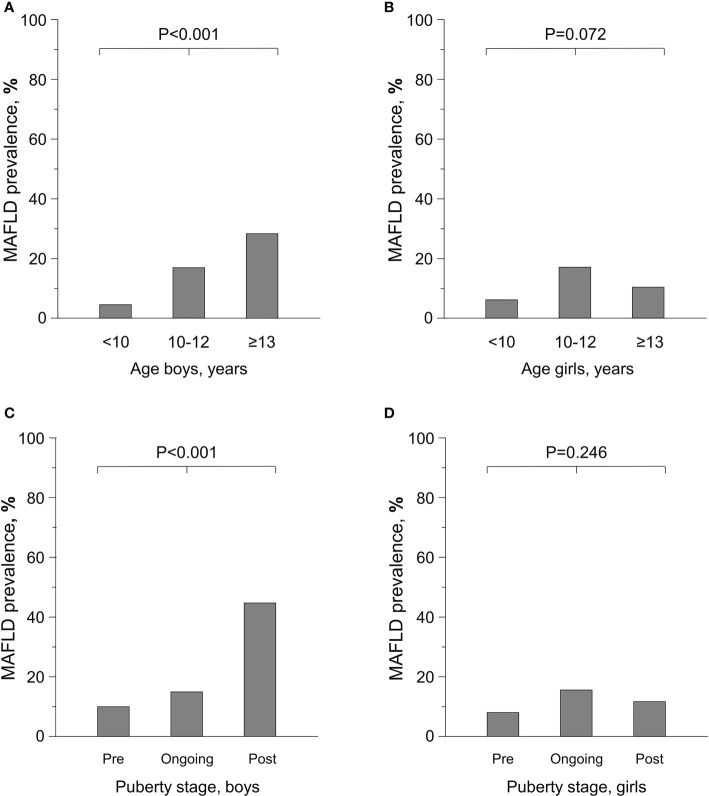
Prevalence of Metabolic-Associated Fatty Liver Disease (MAFLD) depending on age **(A**, **B)** and pubertal stage **(C**,**D)** in overweight and obese boys (N=401) and girls (N=302).

**Figure 2:**
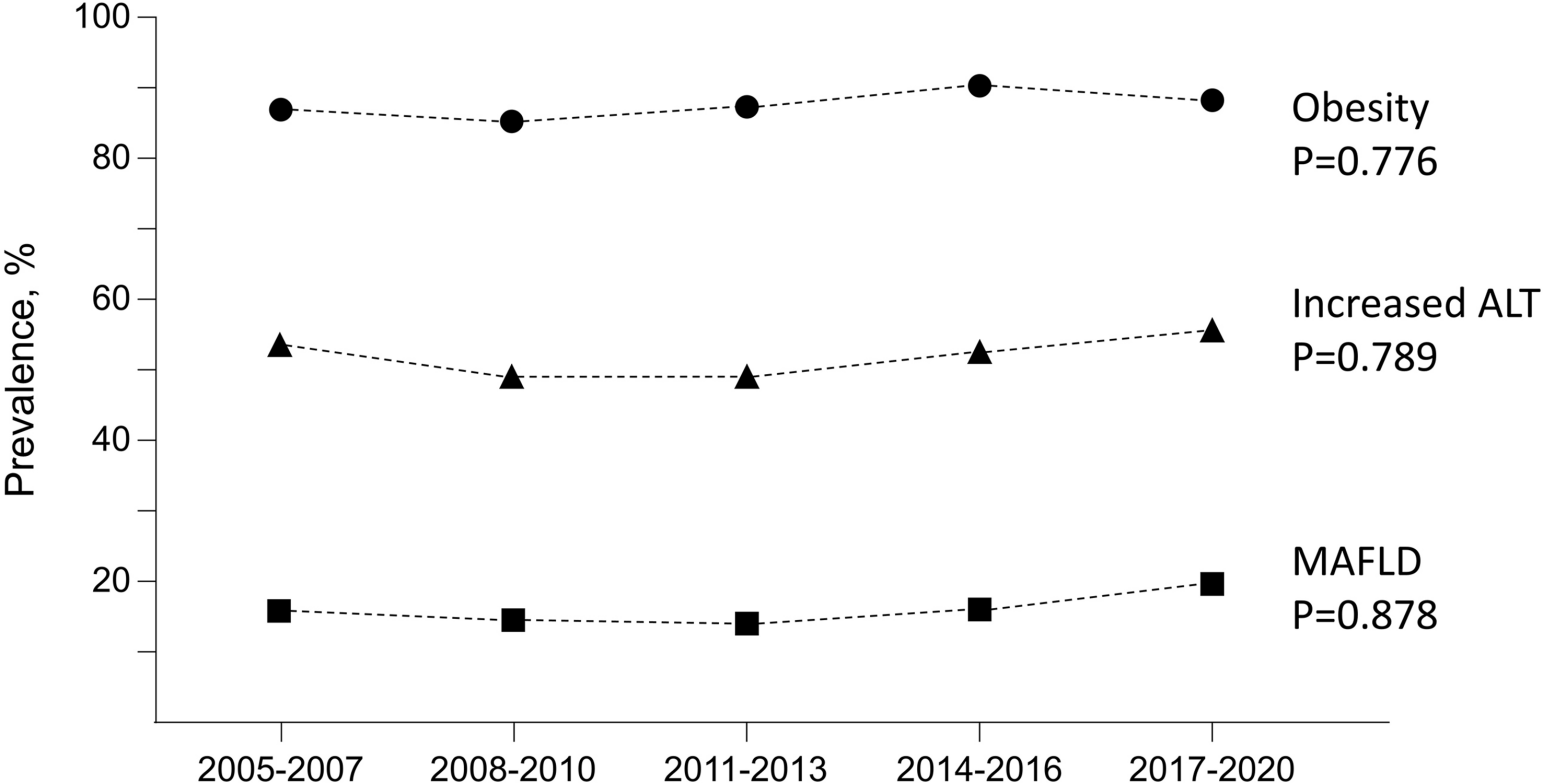
Changes over time in the prevalence of obesity, increased alanine aminotransferase (ALT, reference >22 U/l for girls and 25 U/l for boys) and Metabolic-Associated Fatty Liver Disease (MAFLD) in 659 overweight or obese children and adolescents. Changes in the sex distribution (p=0.771), median ages (p=0.292), median body mass index Z scores (p=0.132) or weight-to-height percentages (p=0.662) during the same period were not significant.

Altogether 51.1% of the children and adolescents presented with increased ALT and 5.0% with ALT >80 U/l. The pooled prevalence of MAFLD over the years was 17.7% in boys, 11.3% in girls and 14.9% in both together. The prevalence increased linearly with age and pubertal stage in boys, whereas in girls it peaked at the age 10-12 years and during early puberty (**Figure 1**). There were no significant changes in the prevalence of obesity, increased ALT or MAFLD during the study period. Prevalence of obesity remained stable over time being 88.4% in 2005-7, 86.3% in 2008-10, 88.8% in 2011-13, 91.5% in 2014-16 and 89.3% in 2017-2020. The corresponding prevalences of increased ALT were 53.7%, 48.7%, 48.4%, 52.5% and 55.4%, respectively, and that of MAFLD 15.8%, 14.5%, 14.0%, 15.6% and 19.6%, respectively (**Figure 2**).

**Figure 3:**
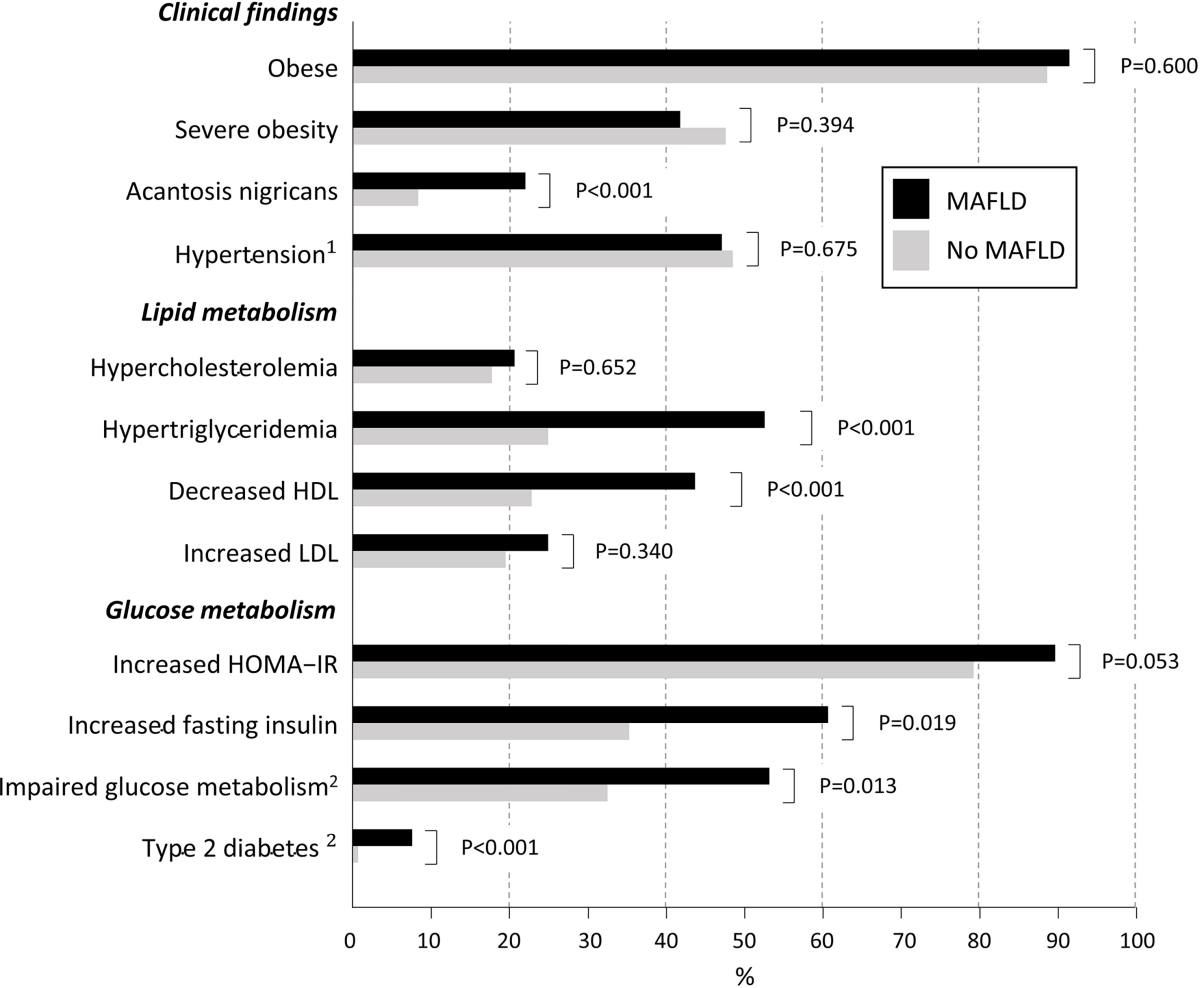
Characteristics of overweight children and adolescents with Metabolic-Associated Fatty Liver Disease (MALFD) (N=105) and without it (N=598). P-values are adjusted for age and sex based on the observed difference between MAFLD and non-MALFD groups in these variables. The following cut-offs for fasting laboratory values were used: total cholesterol ≥5.18 mmol/l; triglycerides ≥1.13 mmol/l (<10 years) and ≥1.47 mmol/l (≥10 years); HDL cholesterol <1.04 mmol/l; LDL cholesterol ≥3.36 mmol/l; HOMA-IR ≥2.67 for prepubertal and ≥5.22 for pubertal boys and ≥2.22 for prepubertal and ≥3.82 for pubertal girls; insulin prepubertal >15 mU/l, pubertal >30 mU/l and postpubertal >20 mU/l; glucose ≥5.6 mmol/l (22, 23). ^1^Blood pressure >95th percentile (19); ^2^Impaired glucose metabolism based on oral glucose tolerance test (OGTT) two-hour value 7.8-11.0 mmol/l or impaired fasting value 5.6-6.9 mmol/L; and type 2 diabetes on values >6.9 mmol/l or >11.0 mmol/l. If OGTT was not available only fasting glucose values were used (24). HDL, high-density lipoprotein cholesterol; HOMA-IR, Homeostatic Model Assessment of Insulin Resistance; LDL, low-density lipoprotein cholesterol.

Children with MAFLD were older (median 12.8 vs. 11.3 years, p<0.001) and more likely boys (67.6% vs. 55.2%, p=0.018) and had higher median ALT values (68 U/l vs. 22 U/l, p<0.001) compared to those without MAFLD, while there was no difference in median BMI Z-scores (2.4 vs. 2.5, p=0.506) (**Supplementary Table**). In addition, they were more likely to have fasting hypertriglyceridemia, low HDL cholesterol, elevated insulin, impaired glucose metabolism, acanthosis nigricans, and T2D, whereas there was no significant difference in the prevalence of obesity or severe obesity, hypertension or other lipid parameters (**Figure 3**; **Supplementary Table**). Overall, children with MAFLD were more likely to have at least one significant cardiometabolic abnormality (hypertension, impaired glucose metabolism, insulin, HOMA-IR or triglycerides or decreased HDL) than did those without MAFLD (82.9% vs 70.6%, p=0.011). The risk of having MAFLD was highest in patients with multiple metabolic abnormalities (OR 1.58, 95% CI 1.34-1.85, p<0.001).

Higher age and BMI, postpuberty, hypertriglyceridemia, decreased HDL cholesterol, hyperinsulinemia, impaired glucose metabolism, and T2D were significantly associated with MAFLD in boys in crude analysis, and hypertriglyceridemia, hyperinsulinemia and impaired glucose metabolism also after adjusting for age (**Table 1**). In girls, hypertriglyceridemia, decreased HDL cholesterol and T2D were associated with MAFLD both in crude analysis and after adjusting for age (**Table 2**).

**Table 1:** Unadjusted and age adjusted analyses for related factors for metabolic-associated fatty liver disease (MAFLD) in 401 overweight or obese boys.

	Odd ratios (95% confidence interval) for MAFLD	
	Unadjusted	Adjusted for age
Age, years	**1.28 (1.15-1.42)**	N/A
BMI, kg/m^2^	**1.01 (1.05-1.15)**	1.05 (0.99-1.11)
Puberty stage		
Prepubertal	1	
Pubertal	1.18 (0.64-2.18)	
Postpubertal	**5.39 (2.26-12.8)**	N/A
^1^Hypertension^2^	0.85 (0.48-1.51)	
^1^Hypercholesterolemia	0.88 (0.43-1.80)	
^1^Hypertriglyceridemia	**2.97 (1.67-5.30)**	**2.77 (1.53-5.01)**
^1^Decreased HDL cholesterol	**2.16 (1.18-3.99)**	1.66 (0.88-3.14)
^1^Increased LDL cholesterol	1.16 (0.60-2.26)	
^1^Increased HOMA-IR	2.99 (0.82-10.9)	
^1^Increased fasting insulin	**3.20 (1.44-7.10)**	**2.59 (1.13-5.96)**
^1^Impaired glucose metabolism^3^	**2.88 (1.64-5.07)**	**2.10 (1.16-3.80)**
^1^Type 2 diabetes^3^	**7.55 (1.23-46.2)**	4.99 (0.80-31.1)

^1^Analysed as binary variable using normal value/no diabetes as the reference; ^2^Blood pressure >95^th^ percentile (19); ^3^Impaired glucose metabolism based on oral glucose tolerance test (OGTT) fasting value 5.6-6.9 mmol/L or two-hour value 7.8-11.0 mmol/l, and type 2 diabetes on values >6.9 mmol/l or >11.0 mmol/l respectively. If OGTT was not available only fasting glucose values were used (24). The following cutoffs for fasting laboratory values were used (22, 23): total cholesterol ≥5.18 mmol/l; triglycerides ≥1.13 mmol/l (age <10 years) and ≥1.47 mmol/l (≥10 years); HDL cholesterol <1.04 mmol/l; LDL cholesterol ≥3.36 mmol/l; HOMA-IR ≥2.67 for prepubertal and ≥5.22 for pubertal boys and ≥2.22 and ≥3.82 for girls, respectively; insulin prepubertal >15 mU/l, pubertal >30 mU/l and postpubertal >20 mU/l; glucose ≥5.6 mmol/l. BMI, body mass index; HDL, high-density lipoprotein; HOMA-IR, Homeostatic Model Assessment of Insulin Resistance; LDL, low-density lipoprotein; Bolded values denote statistical significance.

**Table 2:** Unadjusted and age adjusted analyses for related factors for metabolic-associated fatty liver disease (MAFLD) in 302 overweight or obese girls.

	Odd ratios (95% confidence interval) for MAFLD	
	Unadjusted	Adjusted for age
Age, years	1.07 (0.96-1.19)	
BMI, kg/m^2^	1.03 (0.97-1.09)	
Prepubertal	1	
Pubertal	2.02 (0.88-4.63)	
Postpubertal	1.41 (0.51-3.92)	
^1^Hypertension^2^	1.19 (0.56-2.54)	
^1^Hypercholesterolemia	2.00 (0.81-4.73)	
^1^Hypertriglyceridemia	**4.28 (1.99-9.21)**	**4.19 (1.94-9.06)**
^1^Decreased HDL cholesterol	**4.06 (1.87-8.79)**	**4.07 (1.83-9.07)**
^1^Increased LDL cholesterol	1.77 (0.73-4.25)	
^1^Increased HOMA-IR	1.69 (0.20-14.6)	
^1^Increased fasting insulin	1.95 (0.69-5.45)	
^1^Impaired glucose metabolism^3^	1.56 (0.73-3.33)	
^1^Type 2 diabetes^3^	**18.1 (3.16-103)**	**16.8 (2.88-98.4)**

^1^Analysed as binary variable using normal value/no diabetes as the reference; ^2^Blood pressure >95^th^ percentile (19); ^3^Impaired glucose metabolism based on oral glucose tolerance test (OGTT) fasting value 5.6-6.9 mmol/L or two-hour value 7.8-11.0 mmol/l, and type 2 diabetes on values >6.9 mmol/l or >11.0 mmol/l respectively. If OGTT was not available only fasting glucose values were used (24). The following cutoffs for fasting laboratory values were used (22, 23): total cholesterol ≥5.18 mmol/l; triglycerides ≥1.13 mmol/l (age <10 years) and >1.47 mmol/l (≥10 years); HDL cholesterol <1.04 mmol/l; LDL cholesterol ≥3.36 mmol/l; HOMA-IR ≥2.67 for prepubertal and ≥5.22 for pubertal boys and ≥2.22 and ≥3.82 for girls, respectively; insulin prepubertal >15 mU/l, pubertal >30 mU/l and postpubertal >20 mU/l; glucose ≥5.6 mmol/l. BMI, body mass index; HDL, high-density lipoprotein; HOMA-IR, Homeostatic Model Assessment of Insulin Resistance; LDL, low-density lipoprotein; Bolded values denote statistical significance.

## 4 Discussion

### 4.1 Prevalence of MAFLD

MAFLD was present in 15% of the overweight Finnish children and, more specifically, in 18% of boys and 11% of girls. There are a limited number of comparable studies, but a meta-analysis by Cholongitas et al. (27) reported figures between 11.6% and 81.8% for NAFLD in children monitored in obesity clinics, the pooled prevalence being 32.5% in boys and 15.5% in girls. Only one included study used increased ALT as a diagnostic outcome (28), while the others used ultrasonography, magnetic resonance imaging (MRI), autopsy findings, transient elastography, and fatty liver index (27, 29). Another meta-analysis by Liu et al. reported overall prevalence of MAFLD to be 50.2% in overweight boys and 35.3% in girls with various diagnostic methods, but studies with ALT as an outcome were excluded (29). Additionally, a meta-analysis by Anderson et al. (15) observed a prevalences of increased ALT as a proxy for NAFLD varying between 6.2% and 27.6% in pediatric obesity clinics.

The varying prevalence of NAFLD could be partially due to differing use and performance of the diagnostic methods, as ultrasonography, for instance, has suboptimal accuracy (4, 27, 30) and MRI and liver biopsy are only performed on selected children (3, 4, 15, 31, 32). Although likewise imperfect as a diagnostic tool (33), ALT is a practical and unbiased option for measuring the prevalence of and temporal trends in MAFLD (4, 11, 25, 30, 32). A major challenge, however, is setting cutoffs for ALT (15). For example, we utilized the often-recommended ULNs that are based on data from 12-17-year-old US children with limited consideration for confounders (4, 11, 25). Moreover, it is unclear if these data are representative of other populations (34). The ALT cutoffs used in earlier reports have varied considerably and have rarely been sex-specific (15, 28). Moreover, the studies have often been small and involved heterogenous cohorts and diverse exclusion criteria for other conditions affecting the liver (15, 27, 28). Altogether, more standardized ALT thresholds based on sophisticated diagnostic outcomes (25, 32) and representative cohorts of both obese and non-obese children are called for. Of note, 5% of the children included in our study had ALT > 80 U/l, which has been associated with a significant risk of having advanced liver disease (26).

### 4.2 Patient-related associated factors

In line with earlier research (15, 29, 33–35), the risk of MAFLD was higher in boys than girls. Furthermore, the prevalence of MAFLD increased with age and puberty in boys, while in girls it already peaked in early puberty. These issues have not been studied with similar outcomes but, as an indirect comparison, Putri et al. (35) also found age – although significant in both genders – to be more strongly associated with increased ALT in boys. On contrary, Bussler et al. found ALT to increase at the onset of puberty in both genders and thereafter to decrease in girls (36). Furthermore, Koutny et al. (37) found no clear association between stage of puberty and ALT values. The peak observed in the prevalence of MAFLD at early puberty and the subsequent decrease in girls might be explained by hormonal changes, as female sex hormones have been associated to decreased liver adiposity in women (38). It must also be noted that fixed ALT cutoffs might not be optimal to all puberty stages (36). Additionally, although BMI continued to increase with age in both genders, on the individual level girls may be more prone to active dieting in adolescence e.g. due to peer pressure. Of note, although overweight is both a major risk factor at population level (39) and also a main criterion for MAFLD (11), we found it to be associated with MAFLD only in boys. Logical explanation could be the ceiling effect caused by the high proportion of severe obesity in the study group. Although the complex associations of sex, puberty, and BMI with MAFLD remain to be fully elucidated, present and past findings (40–42) indicate that these individual features may markedly affect the prevalence of MAFLD.

### 4.3 Associations of metabolic disturbances with MAFLD

Approximately half of the study population showed signs of abnormal glucose and lipid metabolism and hypertension. More specifically, T2D, hypertriglyceridemia, and low HDL cholesterol in both genders and hyperinsulinemia and impaired glucose metabolism in boys were associated with MAFLD. Furthermore, the presence of multiple metabolic abnormalities was also associated with MALFD. These results are in general consistent with those of earlier studies on pediatric NAFLD and MAFLD (8, 35, 37, 39). Together with the results of these studies, our observations show that the prevalences of metabolic disturbances are markedly higher than those reported, for example, among children and adolescents in a US population (43). These findings emphasize the metabolic etiology of MAFLD in the majority of cases and support the novel definition of the condition.

### 4.4 Temporal trends in prevalence

We observed no significant change in MAFLD prevalence during the 2000s. The meta-analyses by Anderson et al. and Liu et al. reported similar findings with NAFLD and MAFLD according to the publication years of the studies included (15, 29). However, Cholongitas et al. found the reported prevalences to be higher in 2012–2019 than in 2004–2011 (27). Additionally, a few population-based studies have reported an increase of NAFLD concurrently with obesity (44–46). We observed no change in obesity in overweight children and adolescents, while these data were not reported in other studies. It must, however, be kept in mind that this finding is applicable only to the Finnish population. Interestingly, the results of recent studies suggest that the risk of NAFLD may be increased irrespective of the severity of obesity that may be explained by early-life programming during pregnancy (47, 48), and further studies on this important issue are warranted.

### 4.5 Strengths and limitations

Our main strengths were the large and well-defined study population, availability of comprehensive medical data and consideration of possible confounders. Inclusion of patients seen in primary healthcare reduces the risk of selection bias compared to studies carried out in specialized centers. The retrospective design was a limitation, although this was counterbalanced by the systematic data collection with a pre-tested protocol and the use of unbiased ALT instead of imprecise disease codes (49). Additionally, exclusion of hepatic comorbidities was based on clinical decision-making and some cases may thus have remained unrecognized, although for example viral hepatitis is particularly rare among Finnish children (50). As regards generalizability, while the results may differ in other populations due to the role of individual and environmental factors in the etiology of MAFLD, the use of a simplified and systematic diagnostic outcome improves the comparability. Nevertheless, the fact that most of the participants were of Finnish origin inevitably reduces comparability to other ethnic groups.

### 4.6 Conclusions

To conclude, MAFLD was present in 18% of overweight Finnish boys and 11% of girls, with no significant change seen during the 2000s. The prevalence of MAFLD increased along with age, pubertal stage, and BMI in boys, while in girls it peaked in early puberty. The condition was also strongly associated with metabolic disturbances. The high prevalence of MALFD in overweight and obese children supports their systematic screening to enable early diagnosis. Furthermore, the here identified risk factors for MAFLD help to focus the limited healthcare resources for careful follow-up of high-risk individuals. Future prevalence studies should aim at better standardization of the possible confounders but, simultaneously, consider the effect of individual characteristics in the criteria used to define MAFLD.

## Data availability statement

The datasets presented in this article are not publicly available due to privacy and ethical reasons. Requests to access the datasets should be directed to linnea.aarela@tuni.fi.

## Ethics statement

Ethical review and approval was not required for the study on human participants in accordance with the local legislation and institutional requirements. Written informed consent from the participants’ legal guardian/next of kin was not required to participate in this study in accordance with the national legislation and the institutional requirements.

## Author contributions

HR: study design, data collection and analysis, drafting of the manuscript. LA: study design, data collection and analysis, drafting of the manuscript. LK: study design and critical revision of the manuscript. SL: data collection and critical revision of the manuscript. PH: study design and critical revision of the manuscript. NV: study design and critical revision of the manuscript. HH: study design, statistical analysis, and critical revision of the manuscript. TL: study design and critical revision of the manuscript. KK: study design, study supervision, and critical revision of the manuscript. No writing assistance was received. All authors contributed to the article and approved the submitted version.
