# Supplementary figures and images for: Prevalence and associated factors of metabolic-associated fatty liver disease in overweight Finnish children and adolescents
Source: Front Endocrinol (Lausanne). 2023 Jun 20;14:1090344. doi: 10.3389/fendo.2023.1090344 (PMC10319394; doi:10.3389/fendo.2023.1090344)

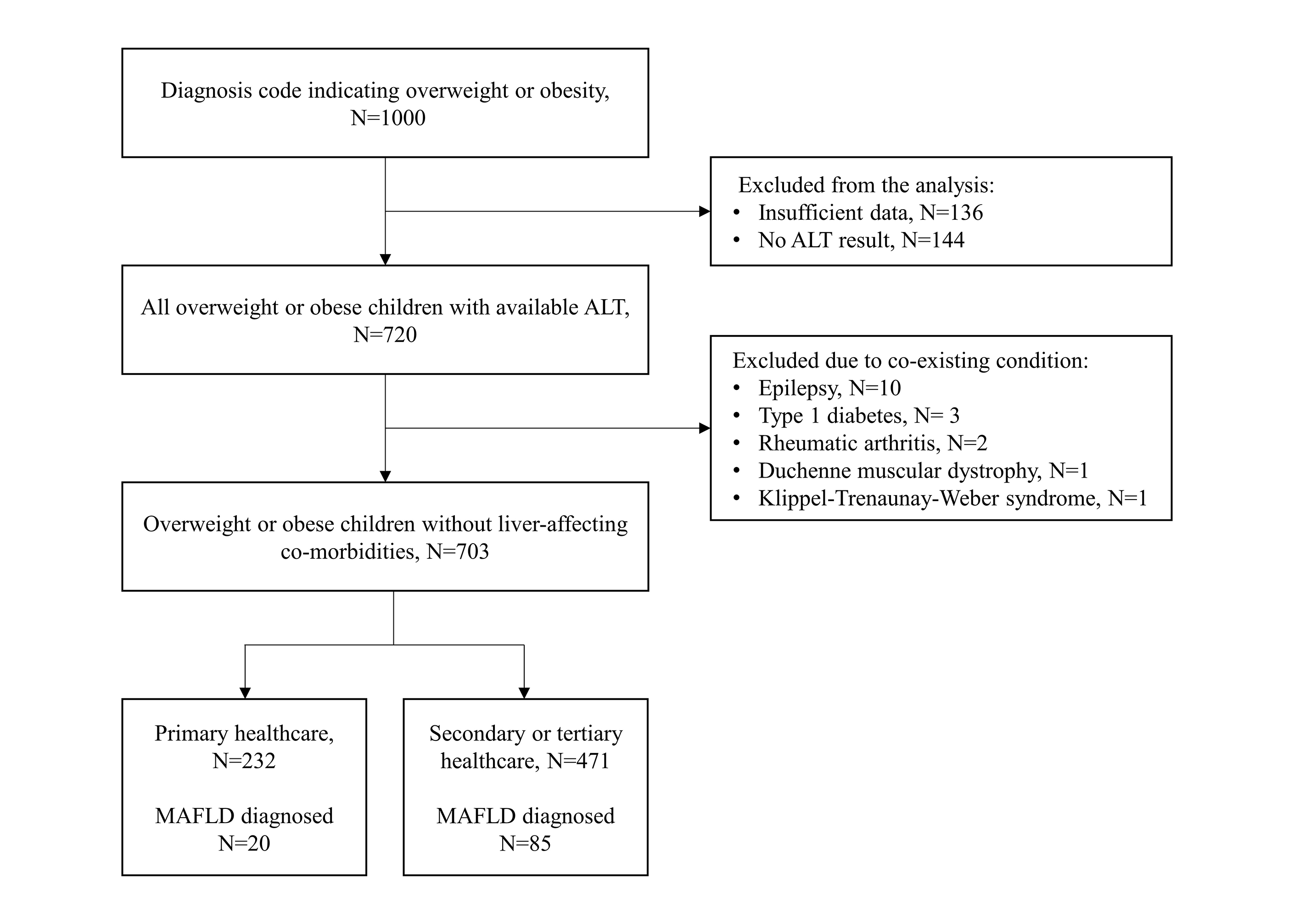

Supplement: Supplementary file 1 [file Image_1.jpeg]
