# Supplementary material for: Prevalence and associated factors of metabolic-associated fatty liver disease in overweight Finnish children and adolescents
Source: Front Endocrinol (Lausanne). 2023 Jun 20;14:1090344. doi: 10.3389/fendo.2023.1090344 (PMC10319394; doi:10.3389/fendo.2023.1090344)
Supplement: Supplementary file 2 [file Table_1.docx]

| **Supplementary Table.** Characteristics of the 703 overweight or obese children and adolescents. | | | | | | | | | |
| --- | --- | --- | --- | --- | --- | --- | --- | --- | --- |
|  | | **MAFLD** | | | | | |  |  |
|  |  | **No, n=598** | | | **Yes, n=105** | | |  |  |
|  | | **Data available** | **Median** | **Q_1_, Q_3_** | **Data available** | **Median** | **Q_1_, Q_3_** | **P value** | **P value^*^** |
| Age, years | | 598 | 11.3 | 8.3, 13.5 | 105 | 12.8 | 10.7,14.4 | **<0.001** | **-** |
| BMI, z-score | | 550 | 2.5 | 2.2, 2.8 | 103 | 2.4 | 2.2, 2.7 | 0.506 | 0.339 |
| ALT, U/l | | 598 | 22 | 17, 29 | 105 | 68 | 55, 97 | **<0.001** | 0.903 |
|  | | **Data available** | **N** | **%** | **Data available** | **N** | **%** |  |  |
| Girls | | 598 | 268 | 44.8 | 105 | 34 | 32.4 | **0.018** | - |
| Obese^1^ | | 598 | 531 | 88.8 | 105 | 96 | 91.4 | 0.423 | 0.600 |
| Severe obesity^1^ | | 598 | 285 | 47.7 | 105 | 44 | 41.9 | 0.276 | 0.394 |
| Central obesity^2^ | | 87 | 86 | 98.9 | 21 | 21 | 100 | 1.000 | 1.000 |
| Hypertension^3^ | | 492 | 239 | 48.6 | 89 | 42 | 47.2 | 0.819 | 0.675 |
|  | Systolic hypertension^3^ | 484 | 200 | 41.3 | 88 | 37 | 42.0 | 0.899 | 0.909 |
|  | Diastolic hypertension^3^ | 488 | 96 | 19.7 | 89 | 14 | 15.7 | 0.384 | 0.468 |
| Acantosis nigricans | | 597 | 50 | 8.4 | 104 | 23 | 22.1 | **<0.001** | **<0.001** |
| **Lipid metabolism** | |  |  |  |  |  |  |  |  |
|  | Hypercholesterolemia | 512 | 91 | 17.8 | 92 | 19 | 20.7 | 0.510 | 0.652 |
|  | Hypertriglyceridemia | 509 | 127 | 25.0 | 91 | 48 | 52.7 | **<0.001** | **<0.001** |
|  | Decreased HDL cholesterol | 507 | 116 | 22.4 | 89 | 39 | 43.8 | **<0.001** | **<0.001** |
|  | Increased LDL cholesterol | 503 | 98 | 19.5 | 88 | 22 | 25.0 | 0.235 | 0.340 |
| **Glucose metabolism** | |  |  |  |  |  |  |  |  |
|  | Increased HOMA-IR | 155 | 123 | 79.4 | 39 | 35 | 89.7 | 0.136 | 0.053 |
|  | Increased insulin | 219 | 80 | 36.5 | 51 | 31 | 60.8 | **0.002** | **0.019** |
|  | Impaired glucose metabolism^4^ | 540 | 176 | 32.6 | 92 | 49 | 53.3 | **<0.001** | **0.013** |
|  | Type 2 diabetes^4^ | 540 | 4 | 0.7 | 92 | 7 | 7.6 | **<0.001** | **<0.001** |
| ^1^As defined by Cole et al.(16) and Saari et al. (17); ^2^Waist circumference >90^th^ percentile (18); ^3^Blood pressure >95^th^ percentile (19); ^4^Impaired glucose metabolism based on oral glucose tolerance test (OGTT) fasting value 5.6-6.9 mmol/L or two-hour value 7.8-11.0 mmol/l, and type 2 diabetes on values >6.9 mmol/l or >11.0 mmol/l respectively. If OGTT was not available only fasting glucose values were used (24). The following cutoffs for fasting laboratory values were used (22, 23): total cholesterol ≥5.18 mmol/l; triglycerides ≥1.13 mmol/l (age <10 years) and ≥1.47 mmol/l (≥10 years); HDL cholesterol <1.04 mmol/l; LDL cholesterol ≥3.36 mmol/l; HOMA-IR ≥2.67 for prepubertal and ≥5.22 for pubertal boys and ≥2.22 and ≥3.82 for girls, respectively; insulin prepubertal >15 mU/l, pubertal >30 mU/l and postpubertal >20 mU/l; glucose ≥5.6 mmol/l. ALT, alanine aminotransferase; BMI, body mass index; HDL, high-density lipoprotein; HOMA-IR, Homeostatic Model Assessment of Insulin Resistance; LDL, low-density lipoprotein. *P value adjusted for age and sex | | | | | | | | | |
